# Recent Studies on Dispersion of Graphene–Polymer Composites

**DOI:** 10.3390/polym13142375

**Published:** 2021-07-20

**Authors:** Suguna Perumal, Raji Atchudan, In Woo Cheong

**Affiliations:** 1 Department of Applied Chemistry, School of Engineering, Kyungpook National University, Daegu 41566, Korea; 2 School of Chemical Engineering, Yeungnam University, Gyeongsan 38541, Korea; atchudanr@yu.ac.kr

**Keywords:** graphene, graphene dispersion, solvents, surfactants, polymers, coating, 3D printing, supercapacitor device

## Abstract

Graphene is an excellent 2D material that has extraordinary properties such as high surface area, electron mobility, conductivity, and high light transmission. Polymer composites are used in many applications in place of polymers. In recent years, the development of stable graphene dispersions with high graphene concentrations has attracted great attention due to their applications in energy, bio-fields, and so forth. Thus, this review essentially discusses the preparation of stable graphene–polymer composites/dispersions. Discussion on existing methods of preparing graphene is included with their merits and demerits. Among existing methods, mechanical exfoliation is widely used for the preparation of stable graphene dispersion, the theoretical background of this method is discussed briefly. Solvents, surfactants, and polymers that are used for dispersing graphene and the factors to be considered while preparing stable graphene dispersions are discussed in detail. Further, the direct applications of stable graphene dispersions are discussed briefly. Finally, a summary and prospects for the development of stable graphene dispersions are proposed.

## 1. Introduction

Graphene is a useful carbon-based material, which is available in nature as graphite. In graphite, single layers of graphene are stacked into many layers by π–π interactions. Graphene is an allotrope of carbon where sp^2^ carbon atoms are arranged in a honeycomb lattice structure [1,2]. Graphene properties have been carefully evaluated since it was separated in 2004 [3]. Graphene is referred to as the strongest and lightest material [4], highly conductive [5,6], and thinnest (adsorbing 2% of light) [7], it has a large specific surface area [8], and is the only one material where reactions can be carried out on both sides [9]. Graphene is used in various fields such as touch panels [10], conducting inks [11,12], flexible electronics [13], electrochemical devices [14], sensing devices [15], and drug carriers [16]. Generally, graphene is prepared by bottom-up and top-down methods as follows:

### 1.1. Chemical Vapor Deposition

The chemical vapor deposition (CVD) process is a straightforward method to prepare graphene, although special types of equipment are needed. In this method, gaseous molecules are deposited on a substrate which are used to grow graphene. The gaseous molecules are combined in the reaction chamber at ambient temperature where they come into contact with the substrate, producing graphene on the substrate [17,18]. The CVD method yields high-quality graphene and the number of layers can be controlled with a homogenous graphene surface. Graphene can be deposited on substrates such as copper [19], nickel [20], platinum [21], and palladium [22]. However, this method has disadvantages: the equipment is expensive, toxic gases are produced as by-products, and it is a sensitive process.

### 1.2. Pyrolysis

Pyrolysis is a solvothermal method used for the chemical synthesis of graphene in a bottom-up process. Thermal reactions of sodium and ethanol are carried out in a closed vessel, graphene sheets were successively detached by sonication [23]. Pyrolysis is a very low-cost process and graphene can be easily fabricated, functionalized graphene can be prepared at low temperatures but the graphene produced by this method has a large number of defects. 

### 1.3. Self-Assembly

Self-assembly is a bottom-up method. Here, graphene is prepared by self-assembling the carbon molecules using sugar derivatives or organic molecules as a carbon source [24,25]. For e.g., polymerization of pyrrole on silica and removing silica layers results in high-quality graphene sheets [26]. The graphene layers can be controlled and it is a simple method. However, large-scale production by this method is difficult.

### 1.4. Thermal Decomposition of Silicon Carbide

Epitaxial graphene on silicon carbide (SiC) is a sublimation process whereby heating SiC to high-temperature results in graphene [27,28]. In this method, silicon atoms are sublimized at high temperatures, leaving behind the graphene layers. This process yields high-quality graphene, however, the SiC wafer itself is expensive and the high temperature required are disadvantages of this method [29,30].

### 1.5. Chemical Exfoliation

The chemical exfoliation method is a top-down method for the synthesis of graphene. Chemical exfoliation includes two steps, the interlayer distance between graphene layers is first increased, then, by using the intercalating compounds, graphites are exfoliated and thus single-layered graphene is produced [31]. Graphene oxide (GO) is prepared by Hummers’ method, where strong oxidizing agents such as potassium permanganate, sulfuric acid, and nitric acid are used to oxidize the graphite into GO [32]. Then GO is reduced by hydrazine [33], sodium borohydride [34], glucose [35], or hydroquinone [36] which produces reduced graphene oxide (rGO). However, in this method strong oxidizing and reducing agents are used. In addition, because of the oxidizing and reducing process, the presence of trace amounts of functional groups on the surface will alter the graphene’s properties.

### 1.6. Mechanical Exfoliation

**Figure 1:**
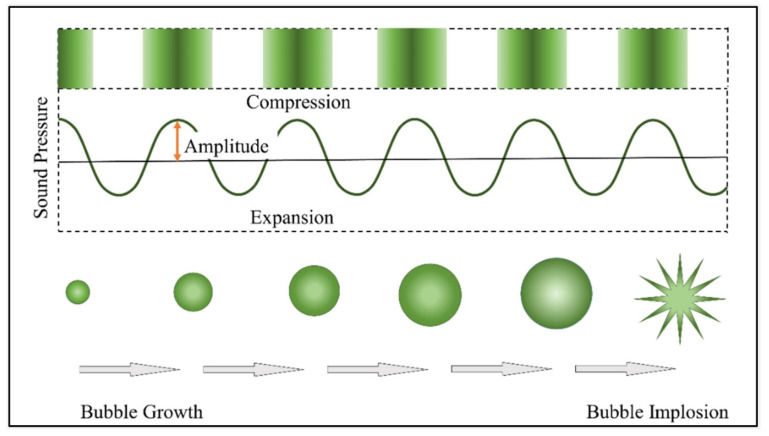
Illustration of the mechanism involved in ultrasonic treatment. Reproduced from Shojaeiarani et al. [40].

Mechanical exfoliation is a top-down method considered as a simple method for the synthesis of graphene. In graphite, graphene layers are stacked by the Van der Waals force, the Van der Waals forces are weakened by sonication, leading to exfoliation. Thus, graphene layers can be prepared from graphite by mechanical exfoliation techniques including micromechanical cleavage using Scotch tape [3], ultrasonication [37], and electric field [38] methods. Exfoliation of graphite using Scotch tape results in multiple layers on the tape. Repeated peeling of the multiple-layered graphene results in graphene with different sizes, ranging from tens of micrometers to nanometers. However, this method requires intensive labor and is time-consuming. Overcoming the attraction between graphene layers will peel the layers from graphite. Lateral (shear force) and normal forces will take place during the exfoliation of graphene from graphite by mechanical methods. Fragmentation, results in graphene of different sizes [39]. Ultrasonication is widely used for the exfoliation of graphite to graphene, this method yields single or few-layered graphene flakes. Figure 1 illustrates the mechanism involved in the sonication process. Sonication involves bath, and horn sonication devices, in these devices electrical energy is transformed to vibrational energy [40,41,42]. The sound waves propagate through the graphene dispersion (liquid medium) in alternating high- and low-pressure cycles. In low pressure cycles, vapor bubbles are formed in the medium and grow, this bubble growth is known as cavitation. The acoustic cavitation bubbles collapse in the high pressure cycles and release strong mechanical and thermal energy which is responsible for the temperature increase in the sonication bath. The release of this energy results in splitting up the larger particles into fine particles and dispersing them in the medium. Additionally, during sonication, insertion of solvent molecules, polymers or intercalation molecules in between the graphene layers takes place which exfoliates the graphite into graphene layers. 

There are many reviews available about graphene, its derivatives, and its composites for different applications [43,44,45,46,47,48,49,50,51,52,53]. However, there is only limited information about the preparation of stable graphene dispersions and the factors to be considered during their preparation. Thus, in this review, the recent advances in graphene dispersions using solvents, surfactants, and polymers are discussed, as well as applications of graphene dispersions.

## 2. Stable Graphene Dispersions Using Solvents 

Graphene is used in various fields; however, many applications need graphene dispersions instead of graphene powders. Graphene flakes, after exfoliation from graphite by ultrasonication, aggregate because of the Van der Waals force between graphene layers [54]. Thus, research has focused on the preparation of stable graphene dispersions. The aggregation of graphene can be overcome by the addition of high-boiling solvents such as N-methyl-pyrrolidone [55] or ortho-dichlorobenzene [56], low-boiling solvents such as chloroform or isopropanol [57], neoteric solvents such as supercritical fluids [57,58], and ionic liquids [59,60]. Graphene dispersions and graphene–polymer composites in organic media have been reported, and these dispersions were stable for the long-term with graphene sheets of a few hundred nanometers in size [61]. However, only specific solvents disperse graphene very well. The graphene dispersibility is dependent on the solvent and graphene properties such as surface tension and solubility parameters (Hildebrand- and Hansen solubility parameters) [62]. The solute–solvent systems divide the intermolecular interactions between the solvent and graphene into three dispersive (D), polar (P), and hydrogen bonding (H) components [63,64,65]. The Hansen solubility parameter calculates the distance of interaction between the solvent and graphene. The smaller distance between the graphene and the solvent, the better the graphene dispersion [66]. Good solvents for graphene should match with Hansen parameters [62] as δ_D_ = 18.0 MPa^1/2^, δ_P_ = 9.3 MPa^1/2^, or δ_H_ = 7.7 MPa^1/2^. The Hildebrand solubility parameters are the sum of D, P, and H Hansen parameters or the square root of the cohesive energy density [62]. Different techniques are used for liquid-phase exfoliation (LPE) of graphite, namely high-shear mixing, sonication, homogenizer, and microfluidization [67,68]. Graphene dispersion in 40 solvents: cyclopentanone, cyclohexanone, *N*-formyl piperidine, vinyl pyrrolidone, 1,3-Dimethyl-2-imidazolidinone, bromobenzene, benzonitrile, *N*-methyl-pyrrolidone (NMP), benzyl benzoate, *N*-*N*′-dimethylpropylene urea, γ-butyrolactone (GBL), dimethyformamide (DMF), N-ethyl-pyrrolidone, dimethylacetamide, cyclohexylpyrrolidone, dimethylsulfoxide (DMSO), dibenzyl ether, isopropylalcohol (IPA), chlorobenzene, 1-octyl-2-pyrrolidone, 1-3 dioxane, ethyl acetate (EtOAc), quinoline, benzaldehyde, ethanolamine, diethyl phthalate, N-dodecyl-2-pyrrolidone, pyridine, dimethyl phthalate, formamide, ethanol (EtOH), vinyl acetate, acetone, water, ethylene glycol, toluene, heptane, hexane, and pentane have been demonstrated [62]. Graphene attains dispersibility of about 8 µg/mL with cyclopentanone as solvent [62]. The studies show that for good graphene dispersion, solvents should have surface tension and Hildebrand solubility parameter of about 40 mJ/m^2^ and 23 Mpa^1/2^, respectively. Solvents that have similar surface tension to graphene will be effective in preparing stable graphene dispersions. The energy required to exfoliate graphite into graphene is balanced by the surface energies of the solvent and graphene [55]. The polar and H-bonding of Hansen parameters of solvent should be nonzero to disperse graphene. 

**Figure 2:**
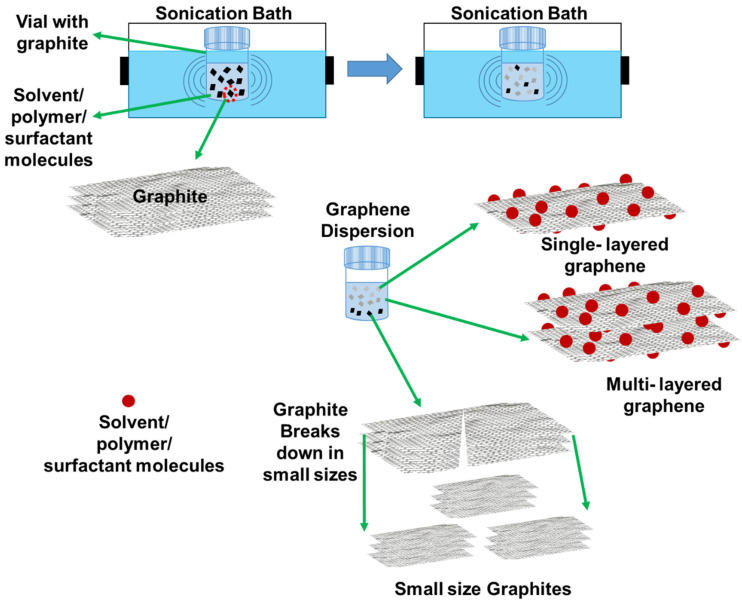
Schematic illustration of the preparation of graphene dispersions using a sonication bath and exfoliation of graphite into graphene.

Other than normal solvents, ionic liquids such as 1-butyl-3-methylimidazolium bis(trifluoromethanesulfonyl)imide and 1-butyl-1-methylpyrrolidinium bis(trifluoromethanesulfonyl)imide, which have surface tensions of about 30 and 40 mN.m^−1^, respectively, showed stable dispersions. Further, these ionic liquids partially exfoliate graphite into single or few-layered graphene [60]. This discussion suggests that choosing a solvent that matches the surface tension and solubility parameters with graphene will effectively produce stable graphene dispersions. Figure 2 illustrates the scheme to prepare graphene dispersions using solvents.

**Figure 3:**
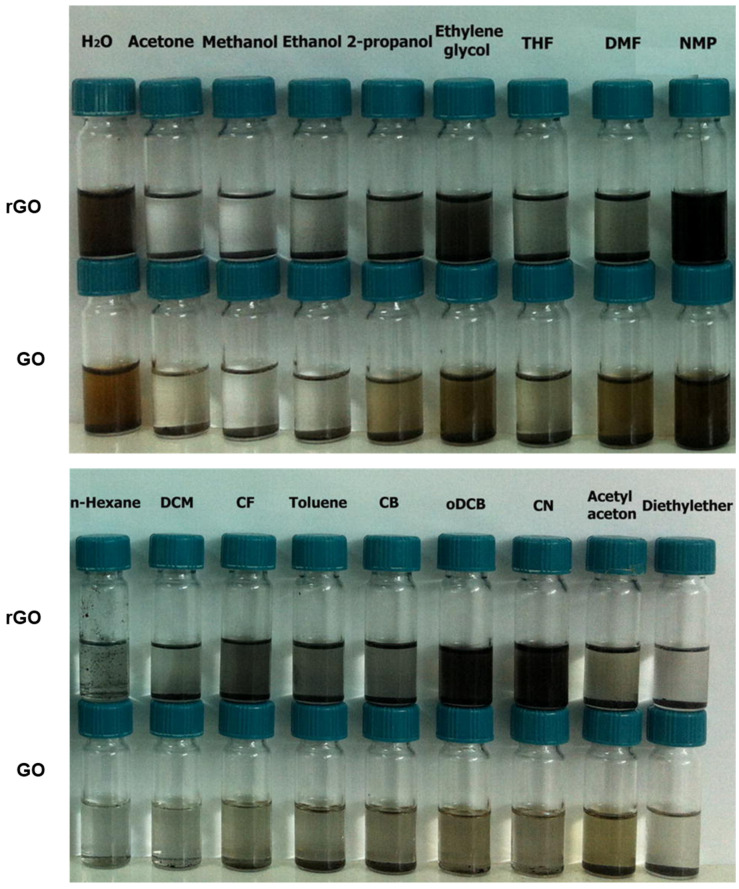
Photographic images of GO and rGO dispersions in different solvents. Reproduced with permission from [74].

Pretreated graphite flakes by tip sonication, and subjected to bath sonication, attained graphene concentrations up to 20 mg/mL. Re-dispersion of pretreated graphene results in good quality graphene dispersions with concentrations of at least 63 mg/mL [69]. Even after 200 h, the concentration of graphene was calculated as 35 mg/mL. Additionally, the dispersions have good quality graphene flakes with an average of three-layered graphene with a lateral size of 1 µm. Ding et al. [70] explained a water-based “green” approach for the preparation of stable aqueous-compatible graphene nanoplatelets. Graphite nanosheets were dispersed into DMF and exfoliated by ball milling, resulting in graphene sheets with three layers and a thickness of about 0.8–1.8 nm [71]. Stable graphene slurries in water were prepared by pretreatment of graphite and exposed to shear effect at 20,000 rpm with a shear dispersing emulsifier [72]. GO showed stable dispersions in the long-term in solvents DMF, NMP, tetrahydrofuran (THF), and ethylene glycol [55]. Stable graphene dispersions were obtained using NMP and GBL solvents by the sonication method [73]. Table 1 shows the preparation of graphene dispersions with graphene concentrations in different solvents by sonication and ball milling methods. Dispersion behaviors of GO and rGO in different solvents were discussed using Hansen and Hildebrand parameters, GO and rGO attained ~9 µg/mL concentration [74]. Figure 3 reveals the long-term stability of GO and rGO dispersions after 2 weeks of preparation in different solvents. GO and rGO dispersions in NMP, water, and ethylene glycol were stable for longer times. The stable dispersions are attributed to the repulsion of GO and rGO sheets [74]. GO dispersions can be prepared via conventional and new methods. In conventional methods, a GO dispersion is prepared directly with solvents: water, methanol (MeOH), EtOH, acetone, THF, EtOAc, and toluene. In new methods, GO dispersions was prepared in a two-step process. In the first step, GO was dispersed in water, then the water was removed by centrifugation. In the second step, GO was re-dispersed in solvents such as water, MeOH, EtOH, acetone, THF, EtOAC, and toluene by sonication [75]. GO used to disperse graphene nanoplatelets in water resulted in stable dispersion [76]. Stable graphene dispersions can be obtained using solvents and the main advantage is retaining graphene properties without modification. However, using solvents includes many disadvantages such as re-aggregation of graphene sheets, low yield of graphene, difficulty in removal of high boiling point solvents, and structural parameters that cannot be altered, restricting the applications. 

## 3. Stable Graphene Dispersions Using Surfactants 

To overcome the difficulties in preparing stable graphene dispersion using solvents, surfactants are used. As discussed in the earlier sections, stable graphene dispersions can be prepared only if the solvent’s surface tension and Hildebrand solubility parameters match graphene’s parameters. To prepare stable graphene dispersions using a solvent that does not match graphene’s parameters, surfactants are added. The electrostatic attraction or intermolecular force between surfactants and graphene helps in stabilizing the graphene surface with surfactants and for the even distribution of graphene in the solution.

**Figure 4:**
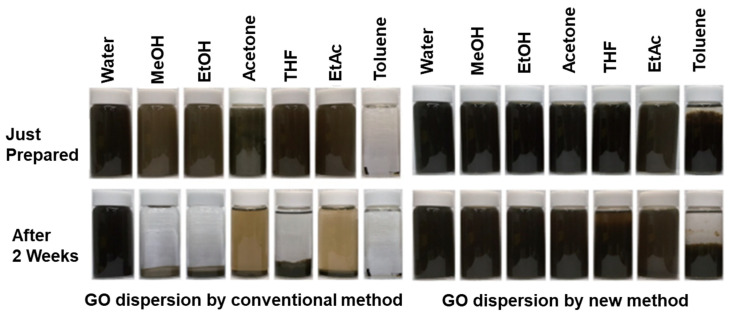
Photographic images of GO dispersions in different solvents prepared by conventional and new methods. Reproduced with permission from [75].

**Figure 5:**
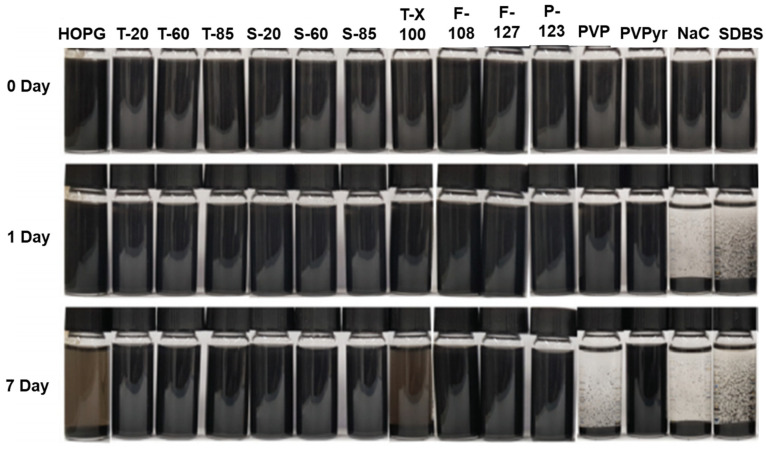
Photographic images of graphene dispersions with different surfactants in NMP. Reproduced from [84]. HOPG: highly pyrolytic graphite; T-20, T-60, and T-85: Tween series; S-20, S-60, and S-85: Span series; T-X100: TritonX-100; F-108, F-127, and P-123: pluronic surfactants; PVP: Poly(4-vinyl pyridine); PVPyr: poly(vinyl pyrrolidone); NaC: sodium cholate; SDBS: sodium dodecyl benzene sulfonate.

Figure 2 illustrates the preparation of graphene dispersions using surfactants and Table 2 shows comparison information of obtained graphene concentrations, surfactants, graphene sources, and dispersion methods. Sodium cholate and Tween 80 disperse graphene at 0.1 and 0.5 mg/mL in water and reach maximum graphene concentration at 10 mg/mL [83]. Graphene dispersions were prepared with 14 different surfactants (Figure 4) in solvents NMP, EtOH, IPA, water, and DCM with a short sonication time (3 h). Stable dispersions were obtained using the Tween series, Span series, and Pluronic surfactants in NMP. In ethanol, nitrogen-based surfactants showed stable graphene dispersions (Figure 5) [84]. Sodium cholate stabilizes the graphene surface and produces high-quality graphene with 1–10 stacked monolayers with lengths and widths of about 1 µm and 400 nm, respectively [85]. Preparing and comparing the stabilities of graphene dispersions in water with surfactants Triton X-100, sodium dodecylbenzene sulfonate (SDBS), and dodecyl trimethyl ammonium bromide (DTAB) were reported [86]. The degree of dispersion using triton X-100 was higher than dispersions using SDBS and DTAB. A novel surfactant from used engine oil was utilized as a dispersing agent for the preparation of stable graphene dispersion [87]. SDBS was used as an intercalating agent to exfoliate graphite into graphene layers and as stabilizing surfactants [88]. Further, the effect of dispersion with the concentrations of SDBS was investigated. Triton X-100 was used to stabilize the graphene surface and exfoliate graphite into graphene using a homogenizer [89]. Aromatic perylene diimide derivatives stabilize and exfoliate graphite into few-layered graphene in the volatile solvent chloroform [90]. Graphite exfoliation into few-layered graphene using cetyltrimethylammonium bromide (CTAB) by a hydrothermal treatment was reported in [91]. 

Highly stable graphene dispersion and exfoliation of graphite were obtained using PVPyr with soap solution [92]. Naphthalene diimide surfactants showed promising results in the exfoliation of graphite and dispersal of graphene in an aqueous solution. The graphene concentrations were calculated as 5 and 1.2 mg/mL by centrifugation at 1000 and 5000 rpm, respectively [93]. Stable graphene dispersions were prepared using surfactants- octadedecyltrimethyl ammonium chloride [94] and anilium dodecylsulphate [95]. Cationic pyrene derivatives were used to prepared stable graphene dispersion by sonicating for 7 days [96]. Graphite was exfoliated and stable GO dispersion was prepared using mixed surfactants—sodium dodecyl sulfate (SDS) and CTAB. The mixed surfactants exhibit better dispersion than the pure ones [97]. rGO stabilization behaviors in water, DMF, EtOH, THF, chloroform, and acetone were studied using SDBS. Chloroform and water showed improved rGO dispersion stability with SDBS [98]. To disperse rGO in water, anionic, non-ionic, and zwitterionic surfactants were used and conditions were varied to obtain stable dispersions [99]. GO is used as a surfactant to exfoliate graphite into graphene [100] and GO is used as a stabilizing agent of graphene nanoplatelets [101].

**Table 2:** A comparison details of dispersion method, graphene source, surfactants, and graphene concentration values.

S. No	Dispersion Method	Graphene Source	Surfactant	Graphene Concentration	References
1.	Sonication	Graphite	Sodium cholate	0.15 mg/mL	[83]
2.	Sonication	Graphite	Tween 80	0.12 mg/mL	[83]
3.	Sonication	Graphite	Sodium cholate	0.3 mg/mL	[85]
5.	Tip Sonication	Graphite	Sodium cholate	7 mg/mL	[65]
6.	Sonication	Graphite powder	Surfactant from engine oil	0.5 mg/mL	[87]
7.	Sonication	Graphite	SDBS	0.05 mg/mL	[88]
8.	Sonication	Graphite micrograins	Sodium cholate	0.52 mg/mL	[102]
9.	Sonication	Graphite micrograins	Sodium deoxycholate	2.58 mg/mL	[102]
10.	Hydrothermal treatment	Graphite powder	CTAB	40–60 µg/mL	[91]
11.	Sonication	rGO	Sodium deoxycholate, poly vinyl pyrrolidone, Briji30	2.3 mg/mL	[99]
12.	Sonication	Graphite	GO	>150 mg/mL	[100]
13.	Sonication	GO	SDBS	1.5 mg/mL	[103]
14.	Sonication	GO	Gallic acid	1.2–4 mg/mL	[104]
15.	Tip sonication	Graphene powders	Silane-based dispersants	10 mg/mL	[105]

Sonication refers to bath sonication.

## 4. Stable Graphene Dispersions Using Polymers 

**Figure 6:**
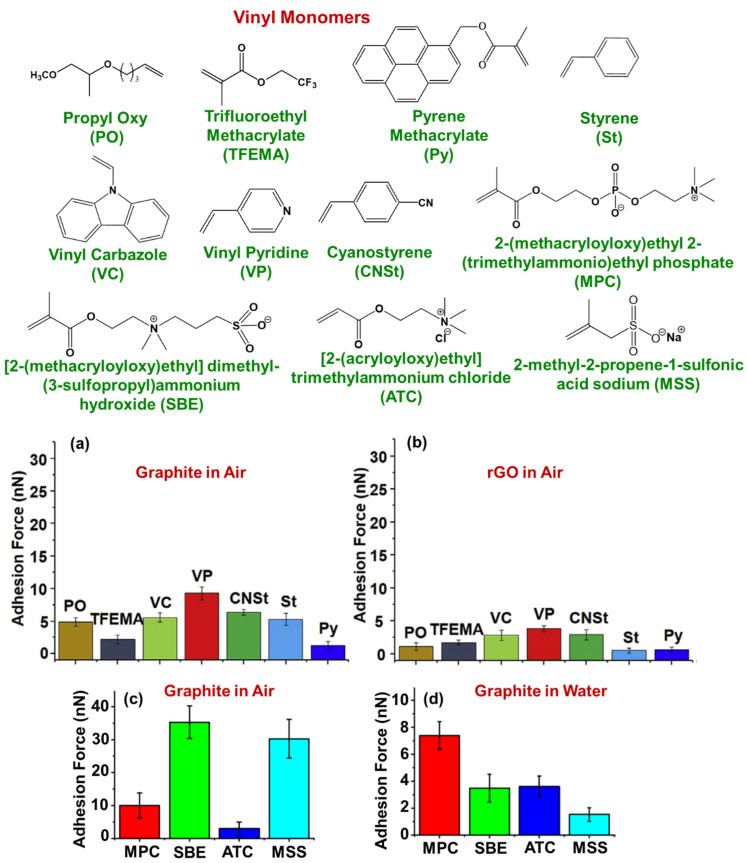
Names and structures of the vinyl monomers used for adhesion force studies of vinyl monomers in air with a graphite surface (**a**) and with an rGO surface (**b**). Adhesion force studies of zwitterion vinyl monomers in air with a graphite surface (**c**) and in water with a graphite surface (**d**). Figures (**a**–**d**) were reproduced from Refs. [114,115] with permission.

In this section, graphene dispersions using polymers are discussed further. As in the earlier sections, factors to be considered for the preparation of stable graphene dispersions using polymers (Figure 2) are discussed. Graphene dispersions using polymers show advantages over dispersions with surfactants and solvents. Polymer functionalization has the advantages of changing molecular weight, topological structure, a choice of polymers appropriate to the application [53,61,106]. Also, polymer functionalization changes the properties of graphene and its composites which are used as components in various applications including energy, film packing, coating, inkjet printing, and in automobiles [11,50,52,107,108,109,110,111,112]. However, achieving stable graphene dispersions using polymers is difficult because choosing a suitable polymer is challenging. This can be overcome if the interaction between graphene surfaces and the stabilizing molecules is known. Recently, we studied the interactions between graphene surfaces and molecules using atomic force microscopy (AFM) [113,114,115]. The AFM cantilevers were modified with various monomers using the hydrosilylation method [116]. Different types of monomers were studied with different graphene surfaces in air and water mediums (Figure 6) [114,115]. Among the different monomers, nitrogen-substituent monomers such as vinyl pyridine showed higher adhesion values with the graphene surface. This is because nitrogen lone-pair electrons interact with the graphene surface along with π–π interactions. Thus, graphene-philic monomers (monomers that showed high adhesion values) were chosen from the AFM studies. 

**Figure 7:**
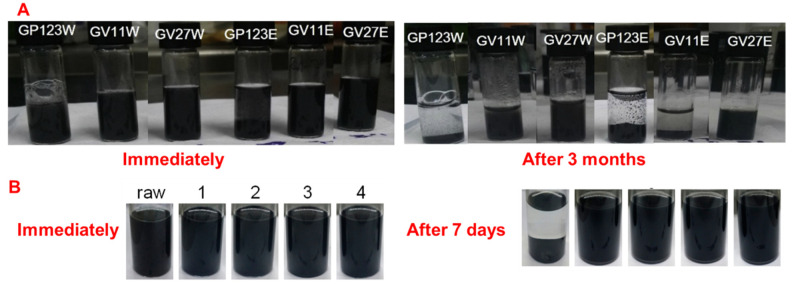
Photographic images of graphene dispersions using PVP-b-PEO in aqueous and EtOH (**A**) and using PTFEMA-b-PVP in MeOH (**B**). (**A**) is reproduced from ref. [113] with permission and (**B**) is reproduced from ref. [118]. G: graphite; P123: P-123 surfactant; W: aqueous medium; E: EtOH medium; V11: PVP(11k)-b-PEO(10k); 27: PVP(27k)-b-PEO(10k); raw: graphite; 1: PTFEMA(11k)-b-PVP(4k); 2: PTFEMA (11k)-b-PVP(21k); 3: PTFEMA(22k)-b-PVP(3k); 4: PTFEMA (22k)-b-PVP(20k).

The polymers poly(4-vinyl pyridine)-block-poly(ethylene oxide) (PVP-b-PEG) and poly(2,2,2-trifluoroethyl methacrylate)-block-poly(4-vinyl pyridine) (PTFEMA-b-PVP) were prepared using chosen monomers (VP showed high adhesion value). Then the prepared polymers (PVP-b-PEG and PTFEMA-b-PVP) were used to disperse graphene in alcoholic and aqueous mediums. As well as dispersing graphene, using these polymers, graphite was partially exfoliated into few-layered graphene [113,117,118,119]. Figure 7 reveals the stable graphene dispersion in alcoholic and aqueous mediums using PVP-b-PEO and PTFEMA-b-PVP. The best solvents among EtOH, NMP, DCM, and THF to disperse graphite using homopolymers and block copolymer-Poly(N-vinyl carbazole) (PVK), poly(4-vinylpyridine) (PVP), and PVK-b-PVP were investigated. DCM was found to be a good solvent to disperse graphene using PVK-b-PVP polymer [120]. Graphite was partially exfoliated and dispersed in water by in situ polymerization on the graphene surface [121]. 

PVPyr with a molecular weight of 10,000 g/mol was used to disperse expanded graphite in different solvents DMF, DMSO, NMP, water, EtOH, and MeOH [122]. PVPyr disperses pretreated graphene by autoclave treatment in different solvents MeOH, EtOH, isopropanol, DMF, DMSO, and NMP [123]. High graphene concentrations of about 3.4 mg/mL were obtained using hyperbranched polyethylene (HBPE) by concentrating the chloroform solvent [124]. The GO surface was well dispersed by in situ polymerization of cyclic butylene terephthalate in solution-free conditions [53]. Stable dispersion of graphene nanoplatelets with a natural polymer of gum Arabic has been reported [125]. GO was dispersed in THF using polystyrene by magnetic stirring, bath sonication, and shear mixing [126]. The presence of colloidal polymer particles at the surfaces of GO and rGO restricts the re-aggregation or restacking of GO and rGO layers [127]. GO dispersed in organic solvents NMP and DMF using polyacrylonitrile and poly(methyl methacrylate) [61]. Stable aqueous rGO dispersions were prepared using conducting polymer poly(3,4-ethylene dioxythiophene):poly(styrene sulfonate) (PEDOT:PSS) [128]. The use of conducting polymers assists the use of graphene composites with PEDOT:PSS in energy storage applications. Table 3 shows the information about graphene concentrations obtained using different polymers.

## 5. Graphene–Polymer Dispersion/Composites Characterization

**Figure 8:**
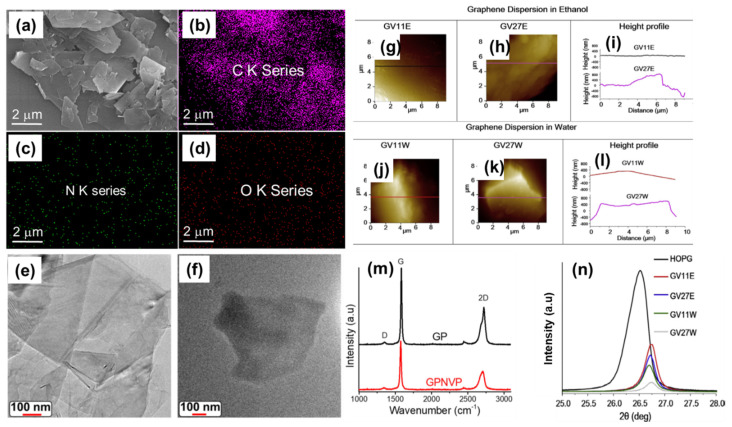
SEM image of graphene-poly(Vinyl pyrrolidone) composite (**a**) and the corresponding elemental mapping of (**b**) carbon, (**c**) nitrogen, and (**d**) oxygen. (**e**,**f**) TEM images of graphene-poly(Vinyl pyrrolidone) composite. (**a**–**f**) images reproduced from ref. [121]. AFM topographic images and corresponding height profiles of graphene–polyvinyl pyridine (11 k and 27 k): (**g**–**i**) ethanolic graphene dispersions and (**j**–**l**) aqueous graphene dispersions. Images (**g**–**l**) reproduced with permission from ref. [113]. Raman spectra of graphite powder and graphene–poly(Vinyl pyrrolidone) composite (**m**), reproduced from ref. [121]. XRD patterns of graphite (HOPG) and graphene–poly(Vinyl pyrrolidone) composites (**n**), reproduced with permission from ref. [113].

Graphene–polymer composites can be studied by several techniques such as transmission electron microscope (TEM), scanning electron microscope (SEM), atomic force microscope (AFM), X-ray diffraction (XRD), Raman spectroscopy, Turbiscan, and thermogravimetric analyses (TGA). Figure 8 depicts the representative SEM, TEM, AFM, Raman, and XRD study results of graphene–polymer composites.

Briefly, TEM and SEM analyses reveal the morphology and size of graphene sheets, and the elements that are present on graphene sheets by elemental mapping. In addition, TEM and SEM studies will suggest the thickness of graphene sheets. As with TEM and SEM analyses, AFM can be used to study the morphology of graphene–polymer composites. The height profile will provide the thickness of graphene sheet from which one can calculate the number of graphene layers. Graphene exfoliated using a mixture of water and alcohol showed graphene sheets with lateral dimensions between several hundred nanometers and micrometers from AFM and TEM measurements. Cross-sections of graphene sheets by TEM showed step heights of ~0.9 and ~0.57 nm suggesting the presence of mono- and bi-layered graphene sheets, respectively [64]. Typical graphene symmetry and atomic ordering of carbon atoms can be observed from selected area electron diffraction–TEM measurements. Further, 75% of flakes of graphene showed a thickness of about 0.9 ± 0.2 nm, and 25% of less than about 3.5 nm thick revealing single- and few-layered graphene sheets, respectively [131]. A Cryo-fractured surface of graphene composites reveals the disordered structure of GO which is attributed to functional groups on the surface and edges [132]. The increase in thickness of the modified graphene surface is due to the presence of molecules/polymers on the graphene surface [103]. In graphene/PVP composites, folded graphene layers from TEM images suggest the presence of graphene two to four layers thick. [122]. The structural aspects can be studied using XRD analysis; a typical pristine graphite peak appearing at 26.5° with an interlayer distance of about 0.33 nm. However, the crystalline nature of graphene will be amorphous in rGO/GO and observed as a broad peak around 25°/9.5°, respectively [114,132]. A decrease in the interlayer distance for graphene composites can be attributed to the presence of excess polymers/surfactants on the graphene surface [113]. Composites prepared by changing the temperature showed an average thickness of about 1.0/1.4 nm which is close to the thickness of single-layered graphene [104]. Raman spectroscopy is a nondestructive powerful analysis for graphene materials. Raman studies will give structural information on graphene materials [132,133,134]. The G band (~1575 cm^−1^) corresponds to the sp^2^ graphitic carbon. The D band (~1355 cm^−1^) is mainly generated due to the breathing mode of aromatic rings from the backbone and the band around 2700 cm^−1^ is referred to as a 2D band, a second order of the D band. Broader G and D bands reflect higher disorder in the graphitic nature. The D band will be barely visible and the 2D band will be sharp in single-layer graphene [114,135,136]. Compared to graphite, in graphene after exfoliation, the G band shifts to a higher position while the 2D band shifts to a lower position [64]. The shift in the G band is important for the exfoliation of graphite into graphene in graphene–PVP composites [122]. The broad G band reveals the defects of GO-ethylene methyl acrylate (EMA) composite which can be attributed to functional groups on the GO surface [132]. Generally, I_D_/I_G_ will be 0.3 for pristine graphene, an increase in this value for composites suggests defects on edge or base [102]. A decrease in I_D_/I_G_ values for GO-SDBS/SDS composites compared to GO suggests a decrease in defects [103]. In order to study the dispersion stability, the multiple light scattering method using Turbiscan Lab instruments has been used [119,137,138]. This method provides the information about the stable dispersions in the upper, middle, and bottom layers of dispersions. The method further reveals any instability such as flocculation, aggregation, or migration of graphene dispersions. An increase in backscattered light intensity with time is attributed to aggregation in the dispersion. TGA is used to characterize the thermal properties of polymers and carbon-based materials. Degradation of polymers takes place step-by-step which helps show the thermal decomposition of the polymer composition as well as giving information about the amount of graphene and polymer present in graphene–polymer composites [139,140,141].

## 6. Graphene–Polymer Composites and Their Properties

**Figure 9:**
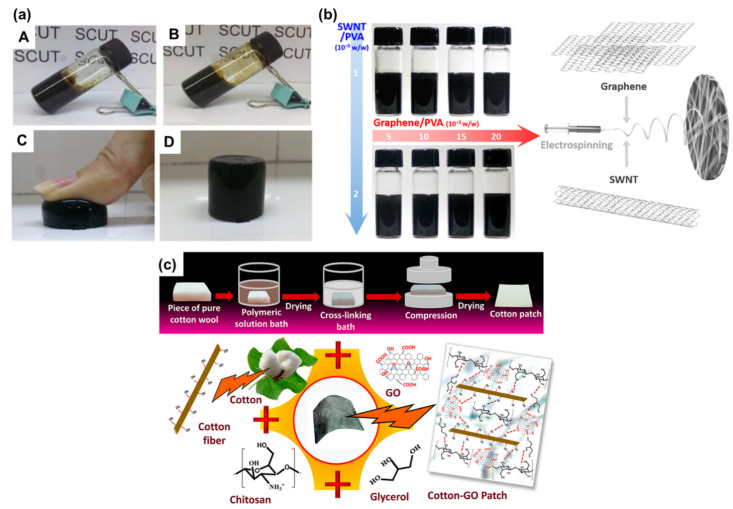
(**a**) GO-NIPAM suspensions, (**A**–**D**) images reproduced with permission from ref. [146], (**b**) Scheme depicts the dispersion of SWNT/graphene/PVA which were electrospun to yield nanofibers, reproduced with permission from ref. [151], and (**c**) schematic representation showing the fabrication of biopolymeric hydrogel coated cotton patches, reproduced with permission from ref. [159].

Graphene–polymer composites improve the properties of pristine polymer materials [142,143,144]. Mainly, the mechanical, electrical, and thermal properties of graphene are increased with polymer inclusion. Graphene–polymer composites include hydrogels, nanofiber, thin films, sponges, hydrocolloids, foams, bandages, and dermal patches. In order to prepare these materials, stable graphene dispersion is a key factor. PAA and chitosan (CS) were dissolved first and then mixed with GO dispersion to prepare porous CS-PAA-GO composites [145]. Thermoresponsive polymer poly(N-isopropyl acrylamide) (PNIPAM) was used to prepare a hydrogel with rGO without initiating pair using hectorite clay (Figure 9) [146]. 3D porous network structures were prepared using polyaniline (PANI) with GO sheets [147]. The viscoelastic properties of GO are improved by the addition of polyvinylalcohol (PVA) which is due to the hydrogen bonding between GO and PVA chains [148]. The conductivity of rGO-PNIPAM–hectorite clay showed high conductivity of about 6.5 × 10^−3^ S/cm compared to rGO alone [146]. pH- and temperature-responsive semi-interpenetrating hydrogels are reported using GO as a cross linker, NIPAM as a monomer, and sodium alginate as an additive [149]. Nanofibers prepared using graphene–polymer composites that are produced by electrospinning have a high specific area and exhibit improved electronic properties over graphene [150]. 1D single-walled carbon nanotubes and 2D graphene pieces were used along with polymers to produce nanofibers with excellent microstructural and electrical properties (Figure 9) [151]. Graphene–PVA, nanofibers with hundreds of nm and lengths in tens of mm with excellent uniformity and surface smoothness were prepared with liquid-phase exfoliated graphene flakes [152]. Freestanding nanofibers have been prepared using poly(vinyl acetate)–graphene by the electrospinning method [153]. GO modified with cetyltrimethylammonium chloride surfactant is used to improve the dispersity of GO in polyacrylonitrile solution [154]. Graphene-foams play a great role in solving the problem of restacking graphene sheets in composites [49,155,156]. Skin-mounted patches have been prepared using graphene–polymer composites [157,158]. GO with curcumin has been specially designed to work on infected wounds (Figure 9) [159]. The effect of mechanical properties was studied with graphene dispersions/graphene–epoxy composites. The highly dispersed rGO showed higher glass transition, and improved quasi-static fracture toughness were measured [160]. Compared to the pristine bio-epoxy, graphene–epoxy composites showed high thermal stability and a slight increase in the glass transition temperature [161]. Graphene platelets in polystyrene were prepared by two methods, a one-step process (solution compounding) and a two-step process (solution compounding and subsequent melt compounding). Glass transition temperature increased for composites prepared using the two-step process. Thermal conductivity was enhanced for composites, nonlinear and linear behavior were observed for the composite with increased graphene content [162]. GO–polystyrene composites were prepared by sonication for 30/60 min and shear mixing for 60/20 min and the glass transition temperature was improved for a longer reaction time. Furthermore, use of THF enhanced the thermal and thermomechanical properties of the composites [126]. The effects of pre- and post-dispersion of graphene nanoplatelet–Triton X-100 nanocomposites on electro- and thermo-mechanical properties were reported. Post dispersion treatment showed improvements in electrical and thermal properties of the composites compared to pre dispersion [163]. Dispersion, re-aggregation, and mechanical properties of graphene nanoplatelets in epoxy/hardener were studied. Results showed that temperature and viscosity affect the dispersion of graphene greatly. Mechanical properties are enhanced for uniform graphene epoxy composites over the pristine epoxy [164]. Different sized GO (170 to 2060 nm) was utilized for enhancing the dispersion stability of carbon nanotubes. Larger-sized graphene enhances the electrical and mechanical properties [165]. Bio-based unsaturated polyester–GO composites showed excellent mechanical properties with, tensile strength, modulus, and T_g_ of 43.2 MPa, 2.62 GPa, and 105 °C, respectively [166]. Graphene–CNCs composites showed ultimate thermal conductivity, bursting strength, and tensile strength as 0.136 W/mK, 1.514 MPa, and 25.8 MPa, respectively [167]. Cellulose fiber/PVA/GO composites showed anisotropic microstructures with low densities of about 17.95 mg/cm^3^, porosity of about 98.8%, a contact angle 142° revealing the hydrophobicity of the surface, and adsorption capacity increase to 96 times its own weight [168]. Moreover, the composites showed high strength with compressive stress about 80% with strain 0.22 MPa. Graphene concentrations in poly(vinylidene fluoride-co-hexafluoropropylene)–graphene composites are responsible for the increases in conductivities and elasticity values [169]. Ultra-high in-plane electrical conductivity, in-plane thermal conductivity, and tensile strength were measured at ~4500 S/m, ~26 W/m/K, and ~50 MPa, respectively. Nanofibers of poly(D, L-lactic-co-glycolic acid) with GO enhance the hydrophilicity because of the functional groups in GO, thus the prepared nanocomposites were proposed for biomedical applications such as scaffolds [170]. Aniline was polymerized using surfactant (CTAB) in the presence of graphene resulting in G-PANI composite. This composite showed high conductivity which was attributed to the charge carrier by π–π interactions between the PANI and graphene. A maximum power density of about 0.01795 Wm^−2^ was attained [171]. Li and coworkers constructed a battery with polypyrene (PPy) fiber–rGO which delivered an energy density of 264 mWh g^−1^ [172]. A diameter-controlled fiber using graphene/PPy showed high capacitive performance [173]. Homogenous dispersion of polyamide 6-functionalized graphene nanocomposite in caprolactam was prepared by Friedel–Crafts acylation. This composite showed significant improvement in the mechanical properties; a 29% increase of tensile strength was reached with 0.1 wt% of graphene nanosheets [174]. The composite (GO–EMA) showed an increase in tensile strength from 18% to 63% compared to pure EMA [132]. 

## 7. Applications

**Figure 10:**
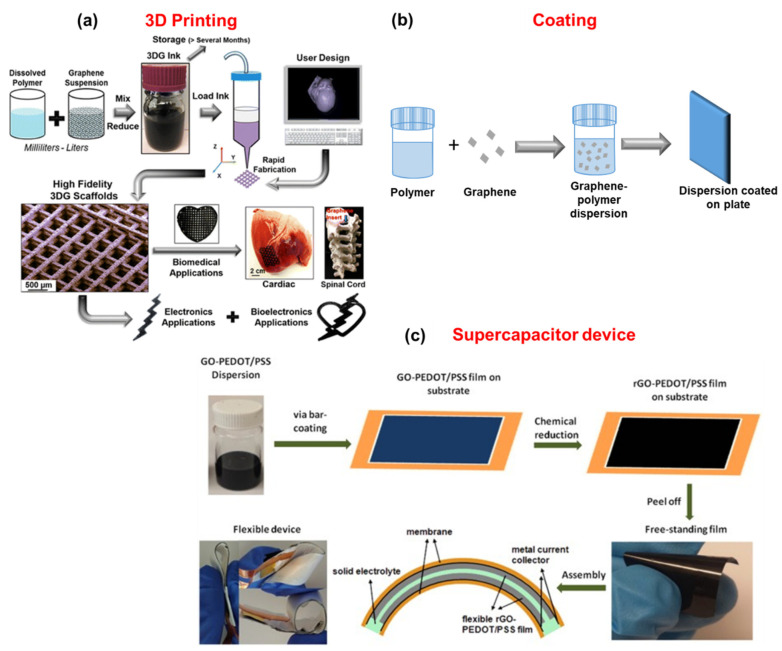
(**a**) Scheme to prepare 3DG ink using graphene dispersion for energy storage and tissue and organ engineering applications, reprinted with permission from [175]. (**b**) Schematic diagram of coating on a substrate using graphene dispersion. (**c**) A graphic illustration of the preparation of GO–PEDOT/PSS film from Go–PEDOT/PSS dispersion and structure assembly of supercapacitor device reproduced from [176].

Many applications of polymer–graphene composites exist, however, only applications such as 3D printing, scaffolds, coating, and supercapacitor are discussed where graphene dispersions are directly utilized (Figure 10).

### 7.1. 3D Printing

3D printing has developed a method of fabricating complex structures that cannot be reached by other methods [177]. 3D printing can be achieved using photopolymerization (stereolithography, material jetting, and two-photon polymerization), extrusion (fused deposition modeling, robocasting), powder-based (selective/selective inhibition laser sintering, selective laser melting, binder jetting, and electron beam melting), laminated object manufacturing, and direct ink techniques [177,178,179]. Graphene–polymer for 3D printing has attracted great attention in biomedical applications, tissue engineering [180], and scaffolds [180,181,182,183,184]. Bioprinting has two types, the pre-seeding or direct method, and the post-seeding or indirect method [185]. Graphene dispersions were directly used in 3D computer-designed fashions in printing applications [186]. Graphene inks prepared using polylactide-co-glycolide were used for printing of high-content graphene scaffolds [175]. This graphene scaffold was used for electronic and biomedical applications. GO was employed to improve the mechanical strength of polyetheretherketone (PEEK) with polyvinyl alcohol (PVA) by π–π interactions with the aromatic rings in PEEK and hydrogen bonds between the functional groups in GO and hydroxyl groups in PVA [187]. Thus GO with PEEk/PVA is used in 3D laser scaffold printing for bone regeneration. Thermally reduced GO with polycaprolactone (PCL) has been used in tissue engineering applications [180]. GO–PCL mixture has been used in printing scaffolds as a substituent for bone tissue engineering. The prepared scaffold was favorable for cell proliferation and differentiation [182]. Using the injection process, 3D scaffolds in the form of sticks were prepared with PCL–graphene nanoplatelets. These sticks were proposed as nasal cartilage [183]. Polylactic acid with GO was used to prepare scaffolds and the prepared scaffolds were proposed for bone formation applications [184]. A graphene–polymer resin octet-truss lattice was used for 3D printing using stereolithography techniques [188]. Asymmetrically aligned structure of graphene thermoplastic polyurethane (PU) composites was utilized for 3D printing [189]. Graphene–polybutylene terephthalate composites have also been used for 3D printing [190]. 

### 7.2. Coating 

Graphene coating applications have multiple advantages such as increasing the resistance to oxygen and water, improving electrical and thermal conductivities, increasing the hydrophobicity on the surface, and preventing scratching and abrasion. However, the presence of the polymer along with graphene helps their use in coating applications [191,192,193,194,195]. Graphene dispersion in DMF prepared by the jet cavitation method has been used for coating applications, to coat Kapton which enhances the atomic oxygen erosion resistance [196]. Waterborne polymers with graphene resulted in stable dispersions and are used for coating applications [197]. Graphene nanoplatelet dispersions with polytetrafluoroethylene improve the friction coefficient, wear rate, and adhesion to the substrate [198]. Waterborne graphene dispersions with lignin–OH is reported that this lignin–OH on graphene surface enhances the anticorrosive properties [199]. rGO has been dispersed in waterborne polyesteramide and used as an anti-corrosive coating material for carbon steel strips [200]. Graphene modified with 1,10-phenanthroline-5-amine helps to detect the corrosion of the steel at an early stage by forming a red complex with Fe^2+^ [201]. Graphene with PU reduces the corrosion rate to 1.81 × 10^−5^ mm per year [202]. Graphene–poly(4-vinylpyridine-co-butyl methacrylate) enhances the corrosion resistance of copper by electrophoretic deposition [203]. The anticorrosion properties are enhanced using GO-3-methacryloxypropyltrimethoxysilane/urushiol-formaldehyde polymer coatings [204]. 

### 7.3. Supercapacitors

Polymer–graphene composites are widely used in energy storage applications. Polymers play an important role as binders in preparing electrodes. However, the usual binders such as polyvinylidene fluoride and polytetrafluoroethylene are not conductive which can decrease the energy density of supercapacitors [205]. Thus, conduction polymers such as polypyrrole, PANI, and poly(3,4-ethylenedioxythiophene) (PEDOT) have been used along with binders to increase the energy density of supercapacitors [206]. This increases the interest in preparing graphene-based electrodes for energy applications [111,207,208]. An activated water-based graphene dispersion with graphene concentration of about 20 mg/mL used as an electrode provided a specific capacitance value of 180 F/g at a specific current of 1 A/g [209]. High quality water-dispersed graphene with dopamine was used for energy storage applications [210]. rGO with PANI is used as an electrode material in supercapacitors [211]. Liquid crystalline GO with PEDOT:PSS dispersion was used for the preparation of binder-free supercapacitor electrodes [212]. Using an rGO-PEDOT/PSS dispersion, a highly flexible, stretchable, and conductive film was prepared and a supercapacitor device was constructed by rolling the film [176]. 3D graphene-based composite hydrogel materials have been used as flexible supercapacitor electrodes. This is because of the outstanding properties of graphene [213]. A PANI/GO composite showed a large specific capacity of 648 F g^−1^ at a current density of 0.5 A g^−1^ [147,213]. Hydrogels using conducting polymers PANI, polypyrrole, and poly(3,4-ethylenedioxythiophene) with graphene are used as hydrogel electrodes. The PANI nanofiber hydrogels exhibited capacitance of up to 492 F g^−1^ at a current density of 1 A g^−1^. 

### 7.4. Other Applications

**Figure 11:**
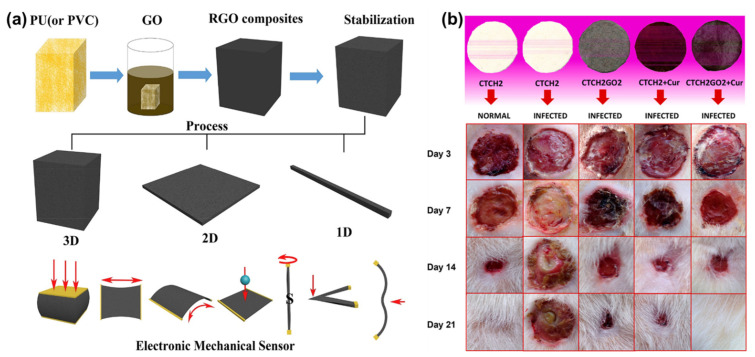
(**a**) Fabrication procedure for rGO/PU sponges and their sensor applications reproduced with permission from ref. [222]. (**b**) Observation of postoperative animal wound healing with different bandage treatments at various time intervals reproduced with permission from ref. [159].

Graphene–polymer can be used to prepare thin films which find applications such as sensors, shielding, field-effect transistors, photodetectors, and gas separation membranes [214]. A thin film was prepared using GO with poly[2,5-bis(3-tetradecylthiophen-2-yl)thieno[3,2-b]thiophene] for an NO_2_ sensor [215]. Graphene nanosheets were dispersed in PU and poly(vinylidene-hexafluoropropylene) to prepare a free-standing conduction thin film which was used for electromagnetic (EMI) inference [216,217]. Thin films from graphene sheets and polystyrene are used for electronics applications [218]. The prepared ultra-smooth surfaces of glassy graphene thin films are used for flexible and transparent circuits [219]. Thin films of PU with graphene affect the recovery behavior of nanocomposites [220]. rGO/CNC sponges were prepared by reducing GO with vitamin C and CNC was used as a stabilizing agent. rGO/CNC has been used for the removal of methylene blue (MB) from water showing an adsorption capacity of 270% [221]. An rGO with PU sponge was used to detect multiple forms of mechanical deformations including tensile strain, impact, bending, vibrating, and twisting (Figure 11) [222]. PU sponges with GO suspension are used as high-performance EMI interference shielding materials. The high shielding effectiveness of 969–1578 dB cm^2^ g^−1^ was observed using a GO/PU sponge [223]. Graphene embedded chitosan, hydroxypropyl cellulose, and polyethylene oxide were used as wound dressings with enhanced antibacterial properties [224]. PEDOT: PSS coated graphene foams have been developed for EMI shielding with a shielding effectiveness of 91.9 dB and specific shielding effectiveness of 3124 dB cm^3^ g-^1^ [225]. Monolayer graphene–polymer has been used for dressing wounds [226]. A smart bandage material was prepared using graphene, CS, and glycerol. These cotton patches without weaving were reported as stable in the presence of an aqueous medium and are highly flexible with excellent mechanical strength (Figure 11) [159]. Ondansetron (ODS) loaded Kapton/rGO was reported as a flexible polyimide-based patch, this patch showed high drug delivery performance on irradiation at 980 nm for 10 min. The release of ODS takes place upon the photothermal heating effect [157]. Electrochemical patches of rGO coated gold nanoholes on Kapton was used for transdermal delivery of insulin [158]. In addition microneedle array patches have been used for the transdermal delivery [227,228,229].

## 8. Conclusions and Future Perspectives

Graphene is used in the multidisciplinary fields and many reviews are available regarding graphene-based composites for various applications. However, there is a lack of information on the preparation of stable graphene dispersions. The preparation of stable graphene dispersions is still under investigation. In this review, the preparation of stable graphene dispersions in different solvents using different graphene sources was presented. The factors to be considered for the preparation of stable graphene dispersion were discussed. For preparing stable graphene dispersion using solvents, solvent’s surface tension and solubility parameters should match with graphene’s surface tension (40 mJ/m^2^) and solubility parameters (Hildebrand solubility parameter of 23 Mpa^1/2^ and Hansen parameters of δ_D_ = 18.0 MPa^1/2^, δ_P_ = 9.3 MPa^1/2^, δ_H_ = 7.7 MPa^1/2^). Surfactants are added to prepare stable graphene dispersions if the solvent’s surface tension and solubility parameters do not match with graphene’s parameters. In stable graphene dispersions using surfactants, electrostatic attraction or intermolecular force between surfactants and graphene surfaces are involved. Graphene with surfactants restricts the applications, thus combining polymers with graphene opens the way for the involvement of graphene dispersions in various applications. To prepare stable graphene dispersion using polymers, the interaction or affinity between polymer molecules and graphene surface should be considered. Thus, in this review adhesion forces between different types of monomers and graphene surfaces were included. The graphene dispersions which are directly used for applications were briefly discussed. This review further opens the way for future perspectives on the factors to be considered while choosing, solvents, surfactants, or polymers to stabilize graphene surfaces. However, further studies are to be concentrated on the investigation of the interaction between stabilizing molecules and graphene surfaces. If we know the interaction between stabilizing molecules and graphene surfaces, we can tune the graphene surfaces with an appropriate stabilizer for particular applications. Therefore, understanding the interactions is very important and this information is lacking. With this understanding, several milestones can be achieved, which will help to design and develop polymers, and molecules to prepare stable graphene dispersions with high graphene concentrations.

## Figures and Tables

**Table 1:** A comparison of dispersion methods, graphene sources, solvents, and obtained graphene concentration values.

S. No	Dispersion Method	Graphene Source	Solvent	Concentration	Reference
1.	Sonication	Graphite	Chloroform	3.4 µg/mL	[62]
2.	Sonication	Graphite	IPA	3.1 µg/mL	[62]
3.	Sonication	Graphite	Acetone	1.2 µg/mL	[62]
4.	Sonication	Graphite	NMP	1.2 mg/mL	[77]
5.	Sonication	Graphene nanoplatelets	Ethylene glycol	0.075 mg/mL	[78]
6.	Sonication	Graphite powder	NMP	2 to 63 mg/mL	[69]
7.	Sonication	Graphite	Actone, chloroform, and isopropanol	0.5 mg/mL	[57]
8.	Ball milling	Graphite nanosheets	NMP, DMF, THF, tetramethyluren (TMU), acetone, ethanol, and formamide	88, 88, 97, 76, 66, 10.32, and 3.67 µg/mL	[79]
9.	Shear mixer (9500 rpm)	Graphite powder	IPA-water mixture	0.27 mg/mL	[80]
10.	Solvent exchange process	Graphite powder	NMP transferred to ethanol	0.04 mg/mL	[81]
11.	Tip sonication	Graphite	Water	0.55 mg/mL	[70]
12.	Sonication	GO	NMP	~8.7 µg/mL	[74]
13.	Sonication	rGO	*o*-dichlorobenzene and chloronapthalene	~9 and ~8.1 µg/mL	[74]
14.	Sonication	Graphite	Water	1 mg/mL	[82]
15.	Pretreatment and shear mixing	graphite	water	50 mg/mL	[72]

Sonication refers to bath sonication.

**Table 3:** A comparison table showing dispersion methods, graphene sources, polymers, and graphene concentration values.

S. No	Dispersion Method	Graphene Source	Polymer	Graphene Concentration	References
1.	Supercritical CO_2_ and Sonication	Graphite	PTFEMA-b-PVP	0.16–0.30 mg/mL	[117]
2.	Sonication	Graphite	PVP-b-PEO	2.6 mg/mL	[113]
3.	Sonication	Graphite	PEO-b-PVP	1.7 mg/mL	[119]
5.	Sonication	rGO	PEO-b-PVP	1.8 mg/mL	[119]
6.	Sonication	Graphite	PTFEMA-b-PVP	0.26–0.38 mg/mL	[117]
7.	Sonication	Graphite	Organosilane	0.66–8.0 mg/mL	[129]
8.	Sonication	Graphite	Polyacryclic acid (PAA)	0.013 mg/mL	[130]
9.	Tip Sonication	Expanded Graphite	PVPyr	0.4–0.72 mg/mL	[122]
10.	Autoclave and sonication	Graphite	PVPyr	0.1 mg/mL	[123]
11.	Heating	GO	PEDOT:PSS	1.0 mg/mL	[128]
12.	Sonication	Graphite	Cellulose nanocrystal (CNCs)	0.3–1.08 mg/mL	[131]
13.	Sonication	Graphite	HBPE in THF	0.016–0.045 mg/mL	[124]
14.	Sonication	Graphite	HBPE in chloroform	0.025–0.18 mg/mL	[124]

Sonication refers to bath sonication.

## Data Availability

No supporting data.
